# Regorafenib: Adding to the Armamentarium for Refractory Colorectal Cancer and GIST

**DOI:** 10.6004/jadpro.2013.4.2.7

**Published:** 2013-03-01

**Authors:** Danielle Roman, Rachelle Whiteside

**Affiliations:** From West Penn Allegheny Oncology Network, Pittsburgh, Pennsylvania

Colorectal cancer is the third most common cancer and the second leading cause of cancer-related mortality in men and women in the United States. The overall lifetime risk of developing colorectal cancer is approximately 1 in 20, with 50,830 deaths expected in 2013 (American Cancer Society, 2013). Despite the frequency of this malignancy, there are limited options for its treatment. Because few patients with metastatic colorectal cancer can be cured by resection, the majority will require treatment with palliative chemotherapy. Treatment often includes conventional chemotherapy such as fluorouracil, capecitabine (Xeloda), oxaliplatin, and/or irinotecan. These agents may be combined with monoclonal antibodies that target vascular endothelial growth factor (VEGF) or epidermal growth factor receptor (EGFR). Patients often exhaust treatment options in early lines of therapy, leaving health-care providers at a loss for additional interventions (Grothey, 2012; Strumberg et al., 2012).

Regorafenib (Stivarga) is a novel oral multikinase inhibitor approved by the US Food and Drug Administration (FDA) for the treatment of metastatic colorectal cancer in patients previously treated with fluoropyrimidine-, oxaliplatin-, and irinotecan-based chemotherapy, anti-VEGF therapy, and EGFR inhibitors (for patients with the wild-type *KRAS* gene). Recently, it was also approved to treat refractory gastrointestinal stromal tumor (GIST) in patients with tumors that cannot be surgically removed and who no longer respond to imatinib and sunitinib (Bayer, 2013).

## Pharmacology and Pharmacokinetics

Regorafenib is an oral diphenylurea-based multikinase inhibitor that is similar to the oral agents sorafenib (Nexavar) and sunitinib (Sutent). Regorafenib is a potent inhibitor of multiple angiogenic and stromal receptor tyrosine kinases, including vascular endothelial growth factor receptor (VEGFR)-1, -2, and -3; platelet-derived growth factor receptor (PDGFR)-beta; PDGFR-alpha; fibroblast growth factor receptor (FGFR)-1 and -2; TIE-2; the proto-oncogene c-Kit; the tyrosine-protein kinase receptor RET; the proto-oncogene RAF-1; BRAF; and MAP kinase p38. Blocking these kinases, which are involved in angiogenesis and intracellular signaling, inhibits tumor growth (Strumberg et al., 2012).

Regorafenib is structurally similar to the multikinase inhibitor sorafenib, but differs by the addition of a fluorine atom in the central phenyl ring (Bayer, 2012). Regorafenib is a type-2 kinase inhibitor, which binds to the inactive kinase, thereby diminishing competition for regorafenib binding by adenosine triphosphate (ATP; Strumberg et al., 2012).

Due to metabolism by CYP3A4, strong inducers and inhibitors of this enzyme should be avoided (Bayer, 2013; see Table 1).

**Table 1:**
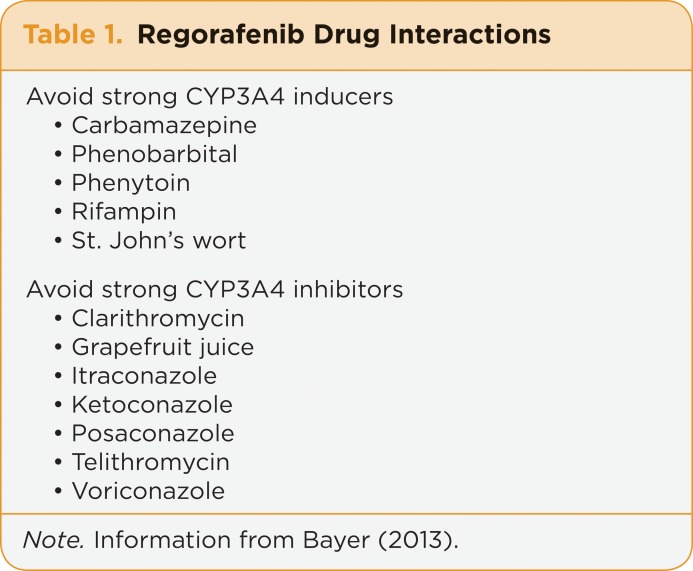
Table 1. Regorafenib Drug Interactions

## Clinical Trials

Regorafenib’s activity was first demonstrated in a phase I trial of patients with advanced solid tumors and progressive disease. The study revealed significant activity in patients with metastatic colorectal cancer and recommended an oral dose of 160 mg daily for a schedule of 21 days on, 7 days off, for future trials (Mross et al., 2012). The phase I trial was further expanded to investigate activity in patients with metastatic colorectal cancer who were heavily pretreated. The results revealed a confirmed partial response in 1 patient (4%), and 19 (70%) other patients had stable disease, for an overall disease control rate of 74%. A total of 13 patients achieved a progression-free response of more than 100 days (Strumberg et al., 2012).

Based on the activity of regorafenib in the phase I trial, a phase III clinical trial (CORRECT) was developed. This trial compared regorafenib plus best supportive care (BSC) with BSC alone. The results were presented at both the 2012 American Society of Clinical Oncology (ASCO) Gastrointestinal Cancers Symposium and the 2012 ASCO annual meeting. The median overall survival (OS) was significantly greater at 6.4 months for regorafenib vs. 5 months for placebo (hazard ratio [HR] = 0.773 [95% CI = 0.635–0.941]; * p* = .0051); in addition, median progression-free survival (PFS) was statistically significantly improved in the regorafenib group compared with the placebo group (1.9 vs. 1.7 months, HR = 0.493 [95% CI = 0.418–0.581]; * p* < .000001). The objective response rates were low at 1.6% with regorafenib and 0.4% with placebo; however, the disease control rate was significantly better with regorafenib (44% vs. 15%; * p* < .000001; Grothey et al., 2012; Van Cutsem et al., 2012).

These results indicate that the primary role of regorafenib is to delay progression and stabilize disease rather than induce a response. A subgroup analysis completed according to region, age, time from diagnosis of metastatic colorectal cancer to randomization, prior lines of treatment, and KRAS status revealed no significant differences in OS or PFS outcomes (Grothey et al., 2012; Van Cutsem et al., 2012). Approval from the FDA was based on the efficacy of regorafenib in this clinical trial (Bayer, 2013).

The use of regorafenib for GIST was first investigated in a phase II trial in patients with intolerance or progression to imatinib and disease progression while on sunitinib. Clinical benefit was seen in 75% of patients with a median of 8 cycles administered. Median PFS was 10 months, and median OS was not reached at the time of publication (George et al., 2012). Secondary to the promising results of the phase II trial, the international phase III GRID trial was created (Demetri et al., 2013). The trial compared oral regorafenib 160 mg daily for a schedule of 21 days on, 7 days off, plus BSC, to BSC alone and included 199 patients that had progressed on both imatinib and sunitinib. Patients were randomized in a 2:1 ratio to regorafenib or placebo. Median PFS was significantly greater with regorafenib at 4.8 months vs. 0.9 months with placebo (HR = 0.27 [95% CI = 0.19–0.39]; * p* < .0001). After progression on placebo, patients were allowed to cross over to the intervention arm; median PFS was 5 months (interquartile range = 3.1–8.7 months). No differences in OS were identified (Demetri et al., 2013). The FDA approval was based on the efficacy of regorafenib in this clinical trial as a third-line therapy option for metastatic or unresectable GIST.

## Dosing, Administration, and Technical Aspects

The recommended dose of regorafenib is 160 mg (four 40-mg tablets) taken once daily for 21 days of each 28-day treatment cycle. Cycles should be repeated until disease progression or the occurrence of unacceptable toxicity. It is recommended that this medication be administered with a low-fat breakfast, which is defined as a meal containing less than 30% fat. Possible low-fat breakfast options include 2 slices of white toast with 1 tablespoon of low-fat margarine and 1 tablespoon of jelly with 8 ounces of skim milk; or 1 cup of cereal, 8 ounces of skim milk, 1 slice of toast with jelly, apple juice, and 1 cup of coffee or tea (Bayer, 2013).

The regorafenib dose does not require modifications in patients with renal or hepatic impairment; however, it has not been studied in patients with severe hepatic impairment (Child-Pugh class C) or in patients with severe renal impairment (creatinine clearance < 30 mL/min/1.73 m^2^) or end-stage renal disease (Bayer, 2013). Due to the potential of impaired wound healing, regorafenib should be discontinued at least 2 weeks prior to elective surgery and may be restarted once surgical wounds are adequately healed (Bayer, 2012a). As mentioned previously, patients taking regorafenib should avoid the strong inducers and inhibitors of CYP3A4 listed in Table 1.

Tablets should be stored at a controlled room temperature in the original bottles; therefore, advanced practitioners should remind patients not to put this medication into a pillbox. Any tablets not used within 28 days of opening a bottle should be discarded (Bayer, 2013).

The average wholesale price of regorafenib is $11,200 for a 21-day supply, or 84 tablets (Thomson Reuters, 2013). Regorafenib is available through select specialty pharmacies. The drug manufacturer has created the REACH (Resources for Expert Assistance and Care Helpline) program to provide support for patients taking regorafenib. Smartphone users can scan the barcode below to access the REACH program; other readers can visit http://www.stivarga-us.com/hcp/mcrc/support.html.

## Managing Adverse Effects

Regorafenib is generally well tolerated, with an adverse effect profile very similar to other small-molecule tyrosine kinase inhibitors with comparable molecular targets, such as sorafenib and sunitinib. Phase I clinical trials identified skin toxicity (hand-foot skin reaction, rash, desquamation, and alopecia) as the most common dose-limiting toxicity. Other common adverse effects identified in clinical trials include fatigue, hypertension, mucositis, diarrhea, and thyroid dysfunction. Regorafenib labeling contains a black box warning for hepatotoxicity due to rare yet fatal occurrences of this adverse effect in clinical trials. Liver function tests should be monitored prior to the initiation of regorafenib, every 2 weeks for the first 2 months of treatment, then monthly as indicated. Should a patient have elevated liver function tests, monitoring should be increased to once weekly until resolution to within three times the upper limit of normal or baseline values (Bayer, 2013).

The majority of adverse effects with regorafenib are mild to moderate and usually straightforward to manage. In the CORRECT trial, ∼8% of patients discontinued the medication due to adverse effects (Grothey et al., 2012). Although specific management guidelines for regorafenib are currently lacking, adverse effect management strategies can be extrapolated from the literature on similar tyrosine kinase inhibitors (Strumberg et al., 2012; Bellmunt, Eisen, Fishman, & Quinn, 2011; Izzedine et al., 2009; Torino et al., 2009).

It is important for advanced practitioners to recognize and manage hand-foot skin reactions that are common in patients taking regorafenib. Nonpharmacologic management strategies can be used for patients with mild cases. Patients should be advised to address preexisting hyperkeratotic areas by using lanolin- or urea-based lotions and having calluses removed from the feet. Cotton gloves and socks with lotion can provide additional protection to skin surfaces. In moderate to severe cases, dose reduction or temporary discontinuation of regorafenib should be considered as described in the package insert (Bayer, 2013).

Diarrhea, seen in 43% of patients on clinical trials (8% grade 3 or 4), can significantly affect a patient’s quality of life. Administering 2 mg oral loperamide 30 minutes prior to each dose of regorafenib is recommended as a preventive measure. If diarrhea develops, the dose of loperamide can be increased to 2 mg after each loose bowel movement. Dietary changes can also provide relief, including foods that are low in fiber (bananas, rice, applesauce, and toast; Bellmunt et al., 2011).

Hypertension is a well-recognized adverse effect of agents that inhibit VEGF signaling. Blood pressure monitoring should be carried out weekly for the first 6 weeks of therapy, then periodically as indicated. The onset of hypertension occurs during the first cycle of treatment for most patients (Bayer, 2013). Due to a lack of controlled studies addressing the management of hypertension in this population, no clear recommendation can be made for a first-line antihypertensive in this setting. Treatment should be initiated as recommended in the 2003 Joint National Committee on Prevention, Detection, Evaluation, and the Treatment of High Blood Pressure (JNC7) guidelines. If possible, verapamil and diltiazem should be avoided, as they are known moderate CYP3A4 inhibitors (Izzedine et al., 2009).

Hypothyroidism is a known complication of several tyrosine kinase inhibitors, including regorafenib. Although the exact mechanism is unknown, it has been postulated that this adverse effect is due to the inhibition of VEGF binding to normal thyroid cells and/or impaired thyroid blood flow, which results in thyroiditis (Torino et al., 2009). It is recommended with similar tyrosine kinase inhibitors that serum thyroid-stimulating hormone (TSH) and free T4 levels be measured with thyroid antibodies at baseline, with a repeat TSH at the start of each new cycle. Elevated TSH levels can be managed with the addition of thyroid replacement; thus, patients typically do not need to discontinue or reduce the dose of regorafenib for this adverse effect (Torino et al., 2009).

## Future Directions

Currently, regorafenib is only approved for the treatment of metastatic colorectal cancer and GIST in the refractory setting. Secondary to regorafenib’s inhibition of multiple key targets as well as efficacy in the refractory setting, its use is now being investigated in the following areas: in combination with chemotherapy as first- and second-line therapy in metastatic colorectal cancer, metastatic renal cell carcinoma, hepatocellular carcinoma, and non–small cell lung cancer (NSCLC).

## FIRST- AND SECOND-LINE THERAPY IN METASTATIC COLORECTAL CANCER

A phase IB study was developed to further investigate the activity of regorafenib in combination with mFOLFOX6 or FOLFIRI as first- or second-line therapy for metastatic colorectal cancer. In the mFOLFOX6 cohort, 19% of patients achieved a partial response and 71% had stable disease; in the FOLFIRI cohort, 24% of patients achieved a partial response and 65% had stable disease (Schultheis et al., 2011). Based on these results, the use of regorafenib in combination with chemotherapy in the second-line setting is now being investigated in phase II trials (National Institutes of Health [NIH], 2013).

## METASTATIC RENAL CELL CARCINOMA

Regorafenib has been shown to inhibit additional pathways compared with sunitinib and sorafenib, which led to the development of a phase II trial to investigate the activity in patients with untreated metastatic or unresectable renal cell carcinoma. The overall response rate was 39.6%, with a median duration of 14.1 months (95% CI = 8.2–17.8 months). All responses were partial. A total of 81% of patients had a clinical benefit (partial responses and stable disease; Eisen et al., 2012). The favorable outcomes and similar toxicity profile of other agents in its class may potentially lead to further investigation and trials in patients with metastatic renal cell carcinoma.

## NON–SMALL CELL LUNG CANCER

A phase I study assessed the safety and efficacy of regorafenib in heavily pretreated NSCLC patients (Kies et al., 2010). Oral regorafenib was given continuously at 100 or 120 mg daily in 23 patients. A total of 17 patients were evaluable for response, and 13 (76%) had stable disease 6 weeks after initiating therapy. Four patients (24%) had progressive disease (Kies et al., 2010). Another phase I trial investigating regorafenib in combination with cisplatin and pemetrexed is underway (NIH, 2012).

## Conclusion

The FDA approval of regorafenib provides another reasonable treatment option for patients with relapsed metastatic colorectal cancer. The National Comprehensive Cancer Network (NCCN) has updated its guidelines to include recommendations for regorafenib as third-line therapy in patients with mutant KRAS and as third- or fourth-line therapy in patients with wild-type KRAS (NCCN, 2013). Regorafenib is also a treatment option for patients with refractory GIST who have previously been treated with imatinib and sunitinib. It is important to remember that the primary efficacy of regorafenib is based on its ability to prevent progression of disease rather than induce a response. In general, patients tolerate treatment well, and adverse effects are easily treated when appropriately managed.

Data currently limit the use of regorafenib to the refractory setting in patients who have failed to respond to all available therapies. However, in the future, use of regorafenib may be expanded to the front-line setting in combination with chemotherapy for metastatic colorectal patients and as a treatment option in other malignancies.
